# Underactive Bladder and Detrusor Underactivity: New Advances and Prospectives

**DOI:** 10.3390/ijms242115517

**Published:** 2023-10-24

**Authors:** Jiaxin Wang, Lida Ren, Xinqi Liu, Jihong Liu, Qing Ling

**Affiliations:** 1 Department of Urology, Tongji Hospital, Tongji Medical College, Huazhong University of Science and Technology, Wuhan 430030, China; jiaxin@hust.edu.cn (J.W.);; 2 Institute of Urology, Tongji Hospital, Tongji Medical College, Huazhong University of Science and Technology, Wuhan 430030, China

**Keywords:** underactive bladder, detrusor underactivity, terminology, etiology, diagnosis, therapy

## Abstract

Underactive bladder (UAB) is a prevalent but under-researched lower urinary tract symptom that typically occurs alongside detrusor underactivity (DU). Unlike UAB, DU is a urodynamic diagnosis which the International Continence Society (ICS) defines as “a contraction of reduced strength and/or duration, resulting in prolonged bladder emptying and/or a failure to achieve complete bladder emptying within a normal time span”. Despite the widespread prevalence of UAB/DU, there are significant gaps in our understanding of its pathophysiological mechanisms, diagnosis, and treatment compared with overactive bladder (OAB) and detrusor overactivity (DO). These gaps are such that clinicians regard UAB/DU as an incurable condition. In recent years, the understanding of UAB has increased. The definition of UAB has been clarified, and the diagnostic criteria for DU have been considered more comprehensively. Meanwhile, a number of non-invasive diagnostic methods have also been reported. Clinical trials involving novel drugs, electrical stimulation, and stem cell therapy have shown promising results. Therefore, this review summarizes recent reports on UAB and DU and highlights the latest advances in their diagnosis and treatment.

## 1. Introduction

Underactive bladder (UAB) is a common lower urinary tract symptom (LUTS) in clinical practice and has received increasing attention from clinicians. Its clinical manifestations are dominated by voiding phase symptoms manifested as a slow stream, intermittency, hesitancy, and feeling of incomplete emptying of the bladder. These may be accompanied by storage phase and post-voiding symptoms. Detrusor underactivity (DU) is a diagnosis based on urodynamics and is defined by the International Continence Society (ICS) as “a contraction of reduced strength and/or duration, resulting in prolonged bladder emptying and/or a failure to achieve complete bladder emptying within a normal time span” [1]. There are numerous causes of DU, including advanced age, neurological disease, low-level spinal cord injury, and bladder outlet obstruction (BOO). According to epidemiological statistics, DU is diagnosed in up to 48% of elderly men and 45% of elderly women undergoing urodynamic evaluations.

Unfortunately, compared to overactive bladder (OAB), BOO, and interstitial cystitis (IC), the study of UAB/DU has been more of a “puddle” in the field of urinary continence. This is due to a combination of factors. First, the absence of a consensus on the definition of UAB has hindered our ability to conduct high-quality epidemiological studies. Such studies provide comprehensive and in-depth descriptions of the natural course and complications of UAB. Second, DU is defined as ICS; however, it lacks recognized diagnostic criteria, and urodynamic testing is not widely available [2]. In contrast, there is a substantial overlap in the clinical presentation of patients with DU and BOO. It is challenging to distinguish between the two without a urodynamic diagnosis, which hampers physicians’ ability to fully recognize DU [3].

Voiding is the coordinated activity of the smooth and transverse muscles of the bladder and outlet (consisting of the bladder neck, urethra, and urethral sphincter) under the control of the nervous system [4]. As the bladder is at the lowest level of innervation, disease or injury anywhere in the voiding reflex (especially the sacral nerve and below) can lead to UAB, which contributes to the pathogenesis of the UAB complex. In the field of basic research, the study of UAB is mostly based on morphological observations and molecular expression and lacks systematic pathway studies, which also makes the development of drug therapeutic targets for UAB slow. According to The European Association of Urology (EAU), intermittent catheterization (IC) remains the preferred treatment for UAB/DU. This procedure involves a patient being catheterized 3–5 times a day to induce urine flow and reduce residual urine [5,6]. However, this technique significantly affects the patient’s typical voiding pattern and quality of life, and may result in complications such as urethral stricture, urethral false channel, hematuria, and bacteriuria [7]. In general, treatment choices for UAB/DU remain inadequate, which considerably hampers the ability of clinicians to treat UAB.

The terminology for UAB was initially standardized and harmonized to facilitate high-level clinical trials. An increasing number of researchers are now trying to find non-invasive diagnostic methods that can help in the early identification of patients affected by UAB. In the therapeutic field, several drugs and stem cell therapies have entered clinical trials with promising results. In addition, attempts to improve the contractility of detrusors using engineering approaches have been reported. Therefore, this review aims to summarize and discuss existing reports related to UAB/DU and to provide an outlook for future research directions.

## 2. Terminology and Definitions

Previously, a variety of terms were used to describe non-obstructive voiding disorders, including impaired detrusor contractility, detrusor areflexia, hypotonic bladder, and detrusor failure. The problems with these terms have been discussed in detail by Osman et al. [8]. Although DU was defined by the ICS as early as 2002, the inaccessibility of DU in primary care limits its application, as it is a urodynamically based diagnosis. The urodynamic features of DU include the absence, low pressure, and/or poorly sustained detrusor contractions accompanied by a low urinary flow rate. However, due to the different etiologies of DU and the age and gender differences in patients with DU, the extent of reduced intensity and the range of “normal time” in the urodynamic definition have not been determined [9]. Therefore, the urodynamic definition of DU remains imprecise, and the term “UAB” has been used incorrectly for a long time. This lack of consistency makes it difficult to conduct high-quality clinical trials and explore potential treatments.

Authorities in the field, including Christopher R. Chapple, proposed a working definition of UAB in 2015 as follows: “underactive bladder is a symptom complex suggestive of detrusor underactivity and is usually characterized by prolonged urination time with or without a sensation of incomplete emptying, usually with hesitancy, reduced sensation on filling, and a slow stream” [3]. In 2018, an ICS working group described UAB as a slow urinary stream, hesitancy, and straining to void, with or without a feeling of incomplete emptying and dribbling, often with storage symptoms” [10]. Both definitions are based on the description and quantification of LUTS without regard to urodynamic findings or specific pathophysiology, which assist primary care physicians in the recognition of the disease. Notably, this symptom-based definition is similar to that of OAB. However, there are effective and universally available treatments for OAB, but not for UAB [9]. Therefore, it cannot be neglected that UAB has a vast array of causes, and effective treatment alternatives that may influence the definition of UAB may arise in the future.

In conclusion, the proposal of standardized terminology for UAB is a pioneering work that is crucial for clinicians to enhance their disease knowledge. Furthermore, we recommend conducting large-scale prospective studies to clarify the precise urodynamic definition of DU based on patient population characteristics. Until sufficient evidence is available, clinical studies should be conducted to accurately and uniformly characterize the natural course of UAB/DU, as suggested by the ICS.

## 3. Epidemiology

The prevalence of UAB in community-based populations is unknown because of the absence of simple, dependable, and non-invasive diagnostic techniques [11]. In 2014, Michael B. Chancellor’s research team carried out a questionnaire survey in Detroit, U.S., and out of the 633 valid contributors (54% males, 46% females), 23% (*n* = 137) reported an emptying disorder of the bladder [12]. At the same time, a higher prevalence of bladder emptying disorders was found in men compared to women (26.1% vs. 20.0%). Additionally, a significantly higher proportion of men over 65 years of age reported bladder emptying disorders (25.5–33.3%). Two years later, the team conducted another study in the USA, referencing the ICS definition of UAB (including straining and feeling of not fully emptying the bladder), and utilized a more comprehensive UAB-q questionnaire [13]. Of the 977 acceptable responses, an increase in reported bladder problems, urinary retention, a past history of catheterization, and frequency of urological visits in the last three years was observed as the frequency of UAB symptoms increased (*p* < 0.0001). Interestingly, there was a positive correlation between the number of UAB symptoms and storage symptoms (prevalence of urgency, frequency, and nocturia) (*p* < 0.0001). Despite the limitations of these reports, which include small sample sizes and possible non-response bias (due to the small number of people who are aware of UAB (11–21%) and due to self-reported difficulties in distinguishing between UAB and other causes of bladder emptying disorders (e.g., BOO)), there is still a considerable value in these initial population-based studies for evaluating the prevalence of UAB. In clinical practice, UAB is frequently associated with underlying diseases. Therefore, discussing the prevalence of UAB independent of its etiology would be unreliable and inaccurate. In the future, large-scale, well-designed, population-based studies that consider relevant etiologies and risk factors will be crucial in determining the incidence of UAB.

The occurrence of DU has been primarily studied retrospectively to distinguish it from LUTS. Osman et al.’s review encompassed multiple retrospective studies and determined that DU rates ranged from 9% to 28% in men aged 18–50, increasing to possibly 48% in males above 70 years of age [8]. Women over a certain age have a DU prevalence ranging from 12% to 45%. A recent systematic review using conventional and imaging urodynamics reported that DU’s occurrence in young men was 24% and 5%, respectively [14]. It is worth noting that the studies included in this review had varying diagnostic criteria for DU, and some did not report specific urodynamic parameters. In contrast, a retrospective study of women attending urogynecology clinics for urodynamic examinations found the prevalence of DU to be between 4.1% to 37.0%, depending on the definition [15]. The various definitions of DU and the non-community origins of the population may place the prevalence of DU over a wide range, making these results informative only for estimating the prevalence of DU.

## 4. Etiology

The voiding process is dependent on two functional units: the bladder (containing the detrusor) and the bladder outlet (containing the bladder neck, sphincter, and urethra). These organs work together to achieve synchronized movements for both storage and voiding processes and are innervated by a complex nervous system involving the peripheral nerves, spinal cord, and higher centers of the brain. Therefore, any organ affecting the voiding reflex may contribute to the development of UAB. The etiology of UAB is unclear; however, it is generally believed to be neurogenic, myogenic, or idiopathic [16]. Osman et al. provided a more detailed categorization of the etiology of DU by adding iatrogenic, functional, and pharmacological categories [17]. Others have suggested that patients with more than two etiologies should be classified into integrative [18] or mixed types [19]. It should be noted that although UAB has been categorized by etiology, patients in a clinical setting often have multiple causes. In this section, we have only discussed the etiology of UAB, as the pathophysiological processes that arise from different etiologies have already been detailed in several existing reviews [2,20,21].

### 4.1. Neurogenic

Neurogenic UAB may arise due to neurological diseases or injury at any site along the voiding reflex, such as the brain, spinal cord, or afferent and efferent nerves. Thus, it is crucial to monitor the lower urinary tract in patients with neurological ailments. The EAU guidelines provide a summary of the established etiologies of neurological disease or injury, classified according to the site and type of lesion into four broad categories, including suprapatine and pontine, the region between the caudal brainstem and sacral spinal cord, the peripheral nervous system, and disseminated central diseases [22]. In terms of overall morbidity, suprapontine and spinal (infrapontine–suprasacral) lesions are generally associated with DO or detrusor–sphincter dyssynergia (DSD), whereas sacral/infrasacral lesions tend to result in DU. However, this relationship is not absolute. During the acute phase of cerebrovascular accidents (stroke), 75% of patients may experience urinary retention due to detrusor areflexia [23]. A recent study examined the frequency of voiding dysfunction in patients with Parkinson’s disease [24]. The study discovered that DU was present in 12% of patients, and an acontractile bladder was present in another 4%. 

Multiple system atrophy (MSA) is a neurodegenerative disease of unknown origin that causes autonomic dysfunction. Clinical manifestations of MSA can be classified into two subtypes: MSA-P and MSA-C. The frequency of DU has been found to be increased to 62.1% in patients with MSA and 71.4% in patients with post-void residual (PVR) > 100 mL. Additionally, the strength of the detrusor in MSA-P has been found to be weaker than that of MSA-C (P_det_@Q_max_ 26.2 vs. 34.4 cmH_2_O, *p* = 0.04) [25]. A recent systematic review found that 12% of patients with multiple sclerosis (MS) experience an atonic bladder [26]. Similarly, a considerable percentage of patients with Guillain–Barre syndrome (GBS) were diagnosed with DU (15/38, 39.5%) [27]. Furthermore, sacral and subsacral nerve injuries have been found to be the leading causes of DU, including cauda equina, lumbar disc herniation, and iatrogenic pelvic nerve injuries. Notably, DU may develop more frequently after rectal resection [28], radical prostatectomy [29], or radical hysterectomy [30].

In addition to these prevalent and acknowledged neurogenic causes, there exist several unusual neurological conditions that could also be contributing to the development of DU, including inflammatory encephalomyelopathy [31], spinocerebellar ataxias (SCAs) [32], Charcot–Marie–Tooth disease (CMT) [33], neuromyelitis optica spectrum disorders (NMOSDs) [34], hereditary spastic paraplegia (HSP) [35], lumbar spinal stenosis [36], idiopathic normal pressure hydrocephalus (iNPH) [37], myelomeningocele (MMC) [38], closed spinal dysraphism (CSD) [39], and Friedreich’s ataxia [40]. Certain neuroviruses, including zoster virus [41] and herpes simplex virus (HSV) [42], may also contribute to the development of UAB. Lee et al. found a higher risk of developing DU in patients with chronic pontine stroke than in those with upper cervical cord injury [43]. Additionally, they identified the central portion of the bilateral pons along the entire sagittal plane as the most frequent site of lesions associated with DU. It has also been reported that the occurrence of OAB or UAB is associated with the site of severe traumatic brain injury, with OAB occurring in left hemisphere injuries and UAB occurring in right hemisphere injuries [44]. Han et al. found that ischemic stroke was associated with a high rate of DO, whereas hemorrhagic stroke was associated with a high rate of DU [45]. This result was consistent with that reported by Burney [23]. Kim et al. concluded that there were no significant differences in lower urinary tract symptoms among patients with dominant, non-dominant, and bilateral hemispheric ischemic strokes [46]. In summary, neurological disorders causing LUTS are multifaceted and diverse, while DU is merely a manifestation. Apart from the level and location of the SCI, no specific type of LUTS has been linked to neurological or psychiatric conditions. Urodynamic testing is essential for the accurate treatment of such patients.

### 4.2. Myogenic

The myogenic etiology of UAB is two-fold: dysfunction of the muscle cells themselves and structural changes in the extracellular matrix due to pathological changes that affect detrusor contraction. In both instances, even with intact nerve reflexes innervating the bladder, the contractile function of the detrusor may be affected. Compared to individuals in normal conditions, patients with DU have approximately four times the amount of “disruptive cells” present in the detrusor. These cells have been characterized by whorled inclusions in the plasma, vacuolation, rupture of the muscle membrane, and enlargement of the cellular gap. Notably, they have not been associated with age or sex, which suggests that there may be factors other than aging that contribute to detrusor deterioration [47]. However, whether alterations in detrusor cells are the cause or consequence of DU requires further research.

It is generally accepted that the main myogenic causes of UAB include BOO and diabetes mellitus (DM, discussed in 4.4). BOO refers to bladder outlet pressure elevation that hinders urination. In such cases, the detrusor attempts to compensate for increased pressure in the bladder through hypertrophy and hyperplasia. However, this compensatory effect is not permanent and continues to progress, leading to a decrease in bladder responsiveness; this is known as the decompensation period [48]. Bladder ischemia and oxidative stress may contribute to myocyte damage [49], whereas inflammation may be implicated in the early compensatory response that leads to fibrosis and, eventually, irreversible extracellular matrix remodeling [50,51].

### 4.3. Idiopathic

Idiopathic UAB primarily comprises UAB related to the aging process and unidentified factors in younger individuals [17]. A common characteristic found among these patients is the lack of neurological disorders or evidence of BOO. The effects of aging on lower urinary tract function are multifaceted. Mature rats have been shown to exhibit extended and weaker bladder contractions than younger and adult rats [52]. In contrast, aging may result in a reduction in the number of 5-HT-positive cells in the urethra, thereby decreasing the excitability of urogenital sensory fibers [53]. It has recently been demonstrated that modified hyperpolarization-activated cyclic nucleotide-gated (HCN) functionality in aged individuals affects their response to autonomic stimuli, inevitably culminating in a reduced capability to maintain bladder flexibility [54]. It has been suggested that metabolic abnormalities may be associated with UAB and DU, rather than aging itself [55]. Therefore, not every elderly person suffers from UAB, although a decline in detrusor contractility may occur in this population, indicating the presence of other underlying causative factors. Moreover, the presence of BOO or other comorbidities in elderly individuals complicates the study of aging as an isolated cause of UAB. However, unknown factors in younger individuals are underreported and warrant further investigation.

### 4.4. Other

Diabetic bladder dysfunction (DSD) is a frequent complication of diabetes mellitus in the urinary tract. The mechanisms that cause DSD to lead to LUTS are intricate, involving both neurogenic and myogenic factors. The progression of the disease follows the initial stages of detrusor overactivity and later stages of detrusor underactivity [56]. Diabetes can cause autonomic neuropathy via axonal degeneration and segmental demyelination [57]. Deficiency of insulin receptors can decrease the downregulation of proteins essential for smooth muscle contraction, such as Chrm3, P2x1, Sm22, and Cav1.2, ultimately leading to reduced smooth muscle contraction [58]. Prolonged diabetes may lead to bladder fibrosis and apoptosis owing to oxidative stress [59]. Klee et al. developed a type 2 diabetes model and discovered that the exogenous administration of carbachol and adenosine triphosphate (ATP) augmented bladder contractility in rats with early stage (1 week) diabetes, indicating a compensatory response [60]. However, after compensation was lost (4 weeks), contractility to ATP increased, whereas contractility to carbachol remained unaltered. Thus, the transition from a compensated to decompensated state may involve a reduction in contractility in response to muscarinic stimuli. Hyperglycemia and osmotic diuresis cause elevated intravesical pressure, leading to compensatory proliferation and hypertrophy of the detrusor. In addition to localized ischemia and oxidative stress in the bladder, this creates an adverse cycle that eventually results in detrusor loss and DU [61].

In addition to anticholinergic drugs that may increase the risk of developing UAB, drugs such as statins [62] and ranolazine [63] may also contribute to UAB development. Although this is a less common issue, clinicians should consider it when making diagnoses.

## 5. Diagnosis

### 5.1. Diagnostic Criteria for DU

As mentioned earlier, although the ICS diagnosis of DU is based on urodynamics, it does not mention specific thresholds to define decreased contraction intensity and prolonged duration of voiding time. Additionally, it does not provide a clear definition of normal time and bladder emptying, which has left the diagnostic criteria for DU unstandardized. Owing to the intricate etiology of DU, a clear urodynamic definition or cutoff value is extremely difficult. Nevertheless, the urodynamic manifestation of DU is a reduction in bladder contractility, and how to accurately estimate bladder contractility is a major concern of researchers. When the bladder contracts, it produces a force that is expressed partly as pressure and partly as flow. As a result, measuring the force of contraction of the bladder using only one parameter is not appropriate. The Watt factor (WF) is a frequently used gauge of detrusor function, which calculates the force per unit area of bladder surface produced by the detrusor, correcting for the limited force required for isometric contraction or no-load shortening [8]. The WF formula is as follows: WF = [(P_det_ + a) × (V_det_ + b) − ab]/2π, where V_det_ depicts the detrusor shortening velocity, and the constants a and b are fixed, obtained from experimental and clinical studies (a = 25 cmH_2_O; b = 6 mm/s). The determination of the projected isovolumetric pressure (PIP) relies on Schafer’s nomogram and is utilized to evaluate the detrusor pressure during isovolumetric contraction of the bladder. The formula for men is PI*p* = P_det_@Q_max_ + 5Q_max_, and a PIP within the range of 100 and 150 is classified as normal contractility [64]. For women, estimating the strength of contraction involves a slightly adapted projected isovolumetric pressure (PIP_1_ = P_det_@Q_max_ + Q_max_) [64]. A PIP_1_ reading between 30 and 75 cmH_2_O is considered normal detrusor contractility. Additionally, another widely employed index, the bladder contractility index (BCI), employs the same formula as PIP. A comparative study has confirmed a good correlation between the above indexes [65].

Based on assessing the strength of bladder contractility, the researchers also considered other indicators to formulate urodynamic diagnostic criteria for DU. The diagnostic criteria for DU are listed in Table 1. Although standardization of the urodynamic diagnostic criteria for DU is lacking, some changes have been observed. Notably, an increasing number of studies have distinguished between definitions for males and females, as females are less susceptible to BOO. The second point to be noted is that the diagnostic criteria are gradually becoming more uniform and are distinguishing between DU and BOO in men. This indicates that clinicians have reached a partial consensus on the urodynamic diagnosis of DU. Recently, the clinical diagnostic criteria proposed by the Japanese Continence Society for DU in men have integrated expert opinions with prior literature reports. These criteria included: (1) UAB symptoms characterized by a slow stream, hesitancy, and difficulty in voiding; (2) a Q_max_ < 12 mL/s and PVR > 100 mL; (3) a bladder voiding efficiency (BVE) < 90%; and (4) a prostate volume (PV) < 30 mL and/or an intravesical prostatic protrusion (IPP) < 10 mm [66]. This criterion displays reasonable sensitivity (37–47%) and substantial specificity (90–94%), facilitating the clinical identification and diagnosis of patients with DU [67]. Jeong et al. compared various diagnostic criteria for men and found that DU was diagnosed in a significantly higher proportion of men with: (1) a BCI < 100 (55.8%) compared to those with (2) a bladder outlet obstruction index (BOOI) < 20 and a free uroflow Q_max_ < 12 mL/s (16.6%), (3) a P_det_@Q_max_ < 30 cmH_2_O and a pressure–flow study (PFS) Q_max_ < 10 mL/s (5.4%), and (4) a BCI < 100, BOOI < 20, and BVE < 90% (10.3%). In addition, they also compared the diagnostic criteria for DU in three groups of women: (1) a Q_max_ < 12 mL/s with ≥100 mL voided or a PVR volume > 150 mL on two or more free flow readings; (2) P_det_@Q_max_ < 30 cmH_2_O and PFS Q_max_ < 10 mL/s; and (3) P_det_@Q_max_ < 20 cmH_2_O, PFS Q_max_ < 15 mL/s, BVE < 90%, and absence of clinical obstruction. The latter two were found to be diagnostic of DU at similar rates (9.6% and 6.4%, *p* = 0.065) [68]. In conclusion, urodynamics is a fundamental test for diagnosing DU, particularly for distinguishing between DU and BOO. Identifying suitable indicators for determining detrusor contractility (such as intensity and duration), bladder outlet resistance, and bladder emptying is a prospective research direction for urodynamic diagnosis.

### 5.2. Non-Invasive Diagnosis of UAB

Given the challenges associated with the widespread use of urodynamics in primary hospitals, including the need for specialized instrumentation and equipment, there has been a growing effort among researchers to develop non-invasive models for diagnosing or predicting UAB. These models are based on a correspondence between signs or symptoms of UAB and urodynamic parameters of DU, or through the use of questionnaires designed to improve the recognition rate of UAB. Gammie et al. conducted a retrospective analysis to identify variations in the symptoms and urodynamics of patients with DU alone and those with DU combined with BOO [83]. This study aimed to determine the differences in symptoms, signs, and urodynamics between the two groups. The findings showed that patients with DU had a lower number of daytime micturition episodes, higher maximal voiding volume, higher prevalence of urinary tract infections (UTI), and greater average urine flow rates and abdominal pressure at maximum flow. The research team conducted a cross-sectional study to compare the distinguishing features of patients diagnosed with DU during urodynamics with those of men who have normal PFS, as well as those with BOO in terms of symptoms, history, bladder diaries, and invasive PFS measurements [76]. Clinical recognition of patients with DU based on their signs and symptoms can be established by the statistically significant incidence of decreased and/or interrupted urine flow, hesitancy, incomplete bladder emptying sensation, palpable bladder, and absent and/or decreased sensation reported by the patients. Chan and colleagues reported that a considerable proportion of patients with nocturia received a diagnosis of detrusor underactivity (DU) alone (*n* = 69, 18.7%) or along with other urodynamic diagnoses (*n* = 108, 29.3%). This suggested that the presence of some other LUTS may be indicative of DU and should be given due attention in the management of the disease [84]. Based on univariate regression analyses, Namitome et al. determined the differences in age, small prostate size, less urgency, weak stream, and low Q_max_ as the main predictors of DU, and developed a predictive model that allows for the non-invasive prediction of DU incidence [80]. Significant parameters between patients with DU and those with BOO were older age (≥74 years old), smaller prostate volume (PV ≤ 34.8), and a lower acute symptom score (IPSS Q4 ≤ 1) [85]. It has also been suggested that DU should be categorized according to the presence or absence of storage phase symptoms, as the proportion of patients with DU combined with storage phase symptoms has been found to be higher in terms of age, prostate volume, and urinary incontinence than that of patients with DU alone [81]. Kim et al. reported a newly developed symptom score-based questionnaire that predicted that a score greater than 45 (the cutoff value) would diagnose a patient with DU with a sensitivity of 95.8% and specificity of 95.4%, which can be used to differentiate between UAB and BOO [86].

Non-invasive uroflowmetry is frequently used to predict the occurrence of DU [87]. Yoldas et al. conducted a retrospective study and discovered that non-invasive uroflowmetry, indicating VE at 46%, had a minimum sensitivity and specificity of 93% and 60%, respectively, for diagnosing UAB in men aged over 80 years [88]. Oelke et al. created a nomogram based on the BOOI and maximum Watts factor, which indicated that DU should be diagnosed in patients below the 25th percentile [89]. Lee et al. concluded that Delta Q (Q_max_ − Q_ave_) can differentiate between DU and BOO in male patients with LUTS [77]. Additionally, it has been suggested that 57% of DU patients have a “sawtooth” curve (defined as having two or more notches) on uroflowmetry examination, compared to 32% of BOO patients [90]. Recently, various research teams have used artificial intelligence (AI) to develop reliable diagnostic methods for DU. Matsukawa et al. determined that the ratio of the first peak flow to Q_max_ in uroflowmetry could accurately diagnose DU based on AI, with a sensitivity of 76%, specificity of 83%, and cutoff value of 0.8 [91]. Bang et al. demonstrated that a finely tuned VGG16 convolutional neural network model could distinguish DU from non-DU [92]. The AI system had a sensitivity and specificity of 79.7% and 88.7%, respectively, for DU diagnosis [93].

Researchers have widely discussed describing detrusor muscle thickness (DMT) for the diagnosis of DU. This is because DU can result from a decrease in the detrusor, which can be identified using ultrasonography; a simple and easily portable technique. However, the use of DWT alone is controversial because of large individual differences in bladder size and the close correlation between DWT and bladder filling. Therefore, recent research has focused on the non-invasive diagnosis of DU using DWT along with other parameters. De Nunzio et al. reported that bladder wall thickness (BWT) and Q_max_ can noninvasively predict DU in a population of patients with LUTS and BPH [94]. Another study reported that ultrasound DWT combined with a bladder capacity of ≥445 mL could be used to diagnose DU in men [95]. The model exhibited a sensitivity of 42%, a specificity of 100%, a positive predictive value of 100%, and a negative predictive value of 42%. Lee et al. revealed that at 20% of the maximum cystometric capacity (MCC), a DMT/BWT ratio of <47.5% was a dependable non-invasive diagnosis of DU [96].

Recently, the PRImary Care Management of Lower Urinary Tract Symptoms (the PriMUS study) in men has been underway, with the aim of providing diagnostic prediction models for the non-invasive identification of LUTS for primary care physicians and ultimately a clinical decision support tool through a validation set [97]. Although several non-invasive diagnostic options have been proposed, it should be noted that the differentiation between DU and BOO still relies on urodynamic testing at the present stage [98]. The diagnostic value of non-invasive diagnostic techniques in DU requires clarification through large-scale prospective clinical studies.

### 5.3. Laboratory Diagnosis

In addition to signs, symptoms, uroflowmetry, and ultrasonography, several research groups have sought laboratory diagnostic methods based on the pathogenesis of DU, including bladder hypoxia, inflammation, and oxidative stress. Jiang et al. identified urinary biomarkers such as 8-hydroxy-2-deoxyguanosine (8-OHdG), prostaglandin E2 (PGE2), epidermal growth factor (EGF), interleukin (IL)-5, IL-8, IL-10, and total antioxidant capacity (TAC) as potential markers for detecting DU [99]. Urinary TAC and PGE2 levels positively correlated with detrusor pressure. Krishnan et al. found that urinary nitric oxide (NO) levels were higher in patients with UAB, whereas adenosine triphosphate (ATP) levels were lower [100]. Therefore, the NO to ATP ratio in urine can potentially serve as a non-invasive diagnostic tool for UAB. A separate study by Ishikawa and colleagues revealed that serum adiponectin levels were significantly lower in patients with DU compared to those without DU (6.2 µg/mL vs. 12.6 µg/mL; *p* < 0.001) [101]. The authors proposed a diagnostic threshold of 7.9 µg/mL, which demonstrated 79% sensitivity and 90% specificity. Majima et al. established a significant positive association between serum albumin, psoas muscle area (PMA), and detrusor contractility [102]. Additionally, certain urinary markers, such as high urinary expression of PGE2 and brain-derived neurotrophic factor (BDNF), can provide insights into the prognosis of patients with DUs, including the likelihood of recovering bladder function [103]. However, these markers related to inflammation and tissue hypoxia do not appear to be DU-specific and require confirmation in large populations while controlling for the absence of other diseases among the participants.

## 6. Therapy

Currently, there is no treatment that can completely cure UAB and significantly enhance patients’ quality of life. As the disease advances, individuals with UAB may experience severe complications, including acute and chronic urine retention, UTI, hydronephrosis, and renal failure, which pose a significant risk to their lives. Therefore, managing UAB primarily involves minimizing residual urine and preventing damage to the upper urinary tract, making etiology-independent conservative treatment the standard option. However, the screening of potential beneficiary populations is also worth further examination with the implementation of new treatment options, including sacral neuromodulation (SNM) and surgery. In addition, UAB treatment is a complex process that requires comprehensive patient education to enhance compliance and improve treatment outcomes. The existing therapies include conservative treatments, pharmacotherapy, electrical stimulation, and surgery. Moreover, novel drugs, such as extracorporeal shock wave therapy (ESWT) and stem cell therapy, have been subjected to multiple clinical trials and have shown significant potential in curing UAB. With remarkable progress in engineering, bladder replacement alternatives have received considerable attention.

### 6.1. Conservative Treatments

Considering the diverse etiologies of UAB, different etiologies may have different patterns of disease progression. UAB secondary to a subsacral spinal cord injury occurs rapidly. The progression of UAB from other causes (e.g., aging and diabetes) tends to be “silent”, making it difficult for patients to detect changes in voiding patterns. Under certain circumstances, patients may experience a shift in bladder function from the OAB (compensated phase) to the UAB (decompensated phase) [104]. Regular checkups and follow-ups are vital in both scenarios. Among these, measuring residual urine is a straightforward yet valuable examination that can reveal BOO or DU. When residual urine levels are high or voiding symptoms are present, interventions should be performed to drain the urine and prevent injury to the upper urinary tract. Conservative treatments involve regular voiding to avoid bladder overdistension and decrease PVR.

The Crede or Valsalva maneuver is the simplest tool-free method for stimulating voiding and reducing PVR. However, these methods often induce reflexive sphincter contractions and elevate the intravesical pressure, thereby increasing the risk of damage to the upper urinary tract [105]. Additionally, employing the Valsalva maneuver in adult spina bifida patients may increase the relative risk of rectal prolapse/intussusception (32.1% vs. 3.7%, *p* = 0.01) [106]. Therefore, prolonged use of these methods for UAB is not advisable.

In light of these concerns, the EAU guidelines suggest intermittent catheterization (IC) as the preferred approach for neurological patients who encounter difficulties in completely voiding their bladders. Ideally, urine should be catheterized 4–6 times per day using a 12–16 Fr disposable catheter, and no more than 400–500 mL of urine should be diverted at any one time. Although IC is considered the gold standard for bladder emptying, its efficacy is greatly limited by patient compliance and tolerance. In addition, IC is inherently linked to UTI and may lead to bothersome complications such as urethral strictures, hematuria, and urethral false passages. Hence, instruction and guidance before IC are essential. Two methods, indwelling transurethral catheterization and suprapubic cystostomy, pose a heightened risk of provoking UTI compared to IC and, therefore, should be avoided as much as possible.

Notably, a randomized clinical trial (RCT) found a significant decrease in PVR and a significant increase in maximum urine flow and voiding frequency following treatment with biofeedback in combination with pelvic floor muscle exercises in patients with non-neurogenic UAB [107]. This investigation included both boys and girls; however, further research is required to determine whether this treatment is effective in adults. In 2014, the US Food and Drug Administration (FDA) approved the InFlow intraurethral valve pump for use in female patients with UAB. A review by Hartigan et al. summarized the use of InFlow for the treatment of UAB in a clinical trial that confirmed the efficacy, low infection rate, and side effects of InFlow; however, it was still not tolerated by approximately half of the patients [108].

### 6.2. Pharmacotherapy

The principle of the pharmacological treatment of UAB mainly includes two aspects: (1) increasing the contractile force of the detrusor and (2) decreasing the resistance of the bladder outlet to promote urine discharge. Notable medications include parasympathetic drugs, alpha receptor blockers, phosphodiesterase type 5 inhibitors, and botulinum toxin A (BoNT/A). Additional pharmaceuticals, such as ASP8302, a selective M3 receptor agonist, and TAC-302, a neuroprotective agent, have been tested in clinical trials. Additionally, the transient receptor potential ion channel subfamily V4 (TRPV4) agonist GSK1016790A has shown promise in improving UAB symptoms in animal studies.

#### 6.2.1. Parasympathomimetics

Acetylcholine (ACh) induces bladder contraction by acting on M3 receptors. As patients with UAB/DU predominantly present with decreased bladder contractility, the application of parasympathetic drugs to enhance bladder contractility may be “theoretically” beneficial. However, the available evidence does not support the use of parasympathomimetic drugs in the treatment of UAB.

The initial parasympathomimetic drugs employed in treating UAB were the non-selective cholinergic receptor agonists bethanechol and carbachol, which are commonly used to treat acute postoperative urinary retention. In 2007, Barendrecht et al. posited that the parasympathomimetic drugs proposed for the management of UAB may be ineffective [109]. This may be due to (1) the poor efficacy of the proposed parasympathomimetic drugs against injuries to the detrusor itself (e.g., bladder wall fibrosis) and (2) the insufficient localized dose to the bladder and the fact that increasing the dose can lead to a range of adverse effects [110]. A meta-analysis that incorporated 12 clinical trials and 3024 patients did not detect any significant differences in mean PVR values. The proposed parasympathomimetic drug had some effect within 1 week (MD −77.5 mL, 95% CI: −90.9 to −64.1, *p* < 0.001), but was not different from the control group at 1 month (MD −10.4 mL, 95% CI −49.7 to 29.0, *p* = 0.6) [111]. Notably, the reported side effects were not as significant as previous literature suggests (odds ratio 1.19, 95% CI 0.62–2.28, *p* = 0.6, moderate quality of evidence). Nevertheless, the available studies are generally of low quality, and the extrapolation of these results is impeded by the inability to perform subgroup analyses, owing to varying patient inclusion criteria and the scarcity of urine flow rate studies. Based on the above evidence, the EAU guidelines strongly discourage the use of parasympathomimetics as a routine treatment for patients with UAB.

Recently, an RCT was used to assess a new positive allosteric modulator of the muscarinic M3 receptor, ASP8302, in patients with UAB. The results demonstrated no significant difference in PVR compared with controls. However, it did increase Q_max_ by 3.8 mL/s (*p* = 0.031) and P_det_@Q_max_ by 2.7 cmH_2_O (*p* = 0.034) [112]. Acotiamide, a new selective inhibitor of acetylcholinesterase (AChE) that impedes the degradation of ACh expelled from the parasympathetic nerve endings and elevates ACh levels in the synaptic gap, has been administered to manage functional dyspepsia. According to Singh et al., acotiamide significantly increases bladder contractility in both rats and humans and may have potential therapeutic effects on UAB/DU [113]. A preliminary single-arm clinical trial revealed that acotiamide effectively reduced PVR in patients (161.4 ± 90.0 mL vs. 116.3 ± 63.1 mL, *p* = 0.006) [114]. Consequently, high-quality clinical trials are necessary to determine the therapeutic potential of proposed parasympathomimetic agents for UAB.

#### 6.2.2. Phosphodiesterase 5 Inhibitors (PDE5is)

PDE5is increase intracellular cyclic guanosine monophosphate by inhibiting phosphodiesterase 5 and are commonly used in the treatment of erectile dysfunction in men. Tadalafil is also becoming the first-line treatment for LUTS with BOO. It has been proven to increase International Prostate Symptom Scores (IPSSs) and International Index of Erectile Function (IIEF) scores, but not Q_max_ [115]. However, it remains unclear whether PDE5 inhibitors can be used as standalone treatments for DU. Recently, Matsukawa et al. conducted a study that found that both tadalafil and silodosin had a significant impact on improving LUTS and voiding function in patients with non-neurogenic DU, and that tadalafil was associated with an improvement in BCI (19.7 ± 17.7 vs. 6.0 ± 12.5, *p* < 0.001) and Q_max_ (3.0 ± 3.0 mL/s vs. 1.7 ± 1.4, *p* < 0.001) [116]. The mechanism of action of tadalafil may be linked to enhanced urethral relaxation [117], increased blood flow to the lower urinary tract [118], improved oxygenation [119], and decreased chronic inflammation [120] of the bladder and prostate.

#### 6.2.3. Novel Drugs

Several drugs have been developed based on the pathogenesis of UAB, and some clinical trials have been conducted (Table 2). TAC-302, a new drug that restores neuronal synaptic growth, has been reported to significantly increase BCI in men and BVE in both sexes, suggesting its potential therapeutic effect in DU [121].

Meanwhile, some drugs have demonstrated efficacy in preclinical trials. Deruyver et al. observed that TRPV4 enhanced bladder function in a rat model of bilateral pelvic nerve injury [122]. Another group demonstrated that the TRPV4 agonist GSK1016790A significantly decreased intercontraction intervals, bladder capacity, voided volume, and PVR [123]. Matsuya et al. reported that the EP2 and EP3 receptor dual agonist ONO-8055 improved bladder function in a monkey UAB model, with an efficacy similar to that of distigmine [124]. Similarly, Sekido et al. found that ONO-8055 significantly reduced the bladder volume, PVR, and voiding pressure in a rat model of lumbar spinal canal stenosis [125]. In addition, Kullmann et al. showed that neuromedin B promotes urination in diabetic rats with UAB through bombesin receptor activation [126]. Chang et al. reported that intravenous injection of a 5-HT(1A) receptor agonist, 8-hydroxy-2-(di-n-propylamino)-tetralin (8-OH-DPAT), effectively improved voiding function in unilateral L5-S2 ventral root avulsion rats [127]. Gratzke et al. showed that cannabinoid (CB) receptor 2 is mainly expressed in the urothelium, bladder sensory nerves, and cholinergic nerves [128]. Application of 3.0 mg/kg CB to rats resulted in a 96% increase in micturition volume (*p* < 0.01) and a 49% increase in flow pressures (*p* < 0.001). Therefore, improving sensory function during urination may also be an effective option.

### 6.3. Electrical Stimulation

#### 6.3.1. Sacral Neuromodulation (SNM)

In 1999, the FDA approved SNM for managing nonobstructive urinary retention, presumably because it helps patients regain voluntary control of their pelvic floor muscles. Over the past two decades, SNM has been effective in various patient populations. Therefore, urologists must carefully identify the patient groups that may benefit from SNM [129,130,131]. Existing studies appear to be contradictory. A multicenter retrospective study reported successful outcomes in 46.6% of patients undergoing SNM, with no significant difference between the sexes [132]. Nevertheless, Coolen et al. reported a markedly higher success rate in women than in men (62% vs. 22%). A record of psychiatric disorders, such as PTSD, enhanced the chances of successful first-stage SNM by 3.92 times in women and 7.71 times in men who had previously undergone transurethral resection of the prostate. Meanwhile, for each 10-year increase in age, SNM success rates decreased by 2.3-fold and 1.3-fold in men and women, respectively [129]. Another study found that patients with detrusor acontractility had significantly lower efficacy when undergoing SNM (*p* = 0.03). Additionally, younger age was a predictor of SNM response (*p* = 0.02). However, sex, a history of neurogenic disease, a previous pelvic surgery, diabetes mellitus, and a history of preoperative micturition had no significant effect on the presence or absence of an SNM response [130]. In conclusion, large-scale multicenter clinical trials are needed to further define populations that could potentially benefit from SNM.

#### 6.3.2. Intravesical Electrical Stimulation (IVES)

IVES improves bladder dysfunction by stimulating the A-delta mechanoreceptor afferent nerves; however, intact afferent nerve circuits and healthy detrusors are required. According to Deng et al., 42 of 89 patients responded to IVES (with a 50% reduction in PVR), while 24 of the 89 patients (with an 80% reduction in PVR) experienced significant symptom improvement [133]. Liao et al. recently conducted an RCT to assess the efficacy of a new electrical bladder stimulation device in patients with UAB [134]. This study revealed that the bladder electrical stimulation device effectively reduced the PVR and increased the Q_max_ and BVE. However, no significant differences were observed in the number of 24-h catheterizations, Patient Perception of Bladder Condition Scale (PPBC-S) scores, or American Urological Association Symptom Index Quality of Life (AUA-SI-QoL) scores between the two groups (Table 3). One of the possible mechanisms of intravesical electrical stimulation for DU treatment is the stimulation of ATP release from the urinary epithelium and the inhibition of NO release [135].

#### 6.3.3. Others

A range of other neuroelectrical stimulation-based methods has been investigated for the treatment of UAB. Kajbafzadeh et al. performed an RCT and found that transcutaneous interferential electrical stimulation (IFES) successfully reduced bladder capacity, shortened voiding time, and improved the proportion of nocturnal bed wetting in pediatric patients with non-neurological UAB (Table 3) [136]. Nonetheless, additional research is needed to assess whether this technique can be generalized to adults. Theisen et al. described that the use of tibial nerve stimulation (TNS) at parameters of 0.5–3 Hz; 1–4 T induced large, sustained bladder contractions in cats, which has the potential to treat UAB [137]. Recently, Wang and colleagues conducted a study showing that repetitive sacral root magnetic stimulation (rSMS) in conjunction with functional bladder exercises significantly decreased the post-void residual (PVR) volume in individuals with spinal cord injuries (from 349.5 ± 58.5 mL to 172.7 ± 44.6 mL) and increased their maximum urine flow rate (from 6.9 ± 3.9 mL/s to 15.7 ± 4.8 mL/s) [138]. Liu et al. reported that electroacupuncture at BL33 significantly improved rat detrusor smooth muscle cell contraction to a greater extent [139].

### 6.4. Extracorporeal Shock Wave Therapy (ESWT)

ESWT has been shown to play a therapeutic role in common urological disorders such as erectile dysfunction, interstitial cystitis, and stress urinary incontinence [140]; however, whether it can be used in the treatment of UAB is unclear. Two preclinical studies demonstrated that ESWT can improve voiding function in female model rats with cryoinjury-induced UAB and may be linked to increased VEGF expression and cell proliferation [141,142]. Another study by Wang et al. revealed that ESWT could potentially ameliorate urinary function in diabetic rats with UAB by restoring neuronal integrity and promoting smooth muscle actin expression [143]. Recently, Shen et al. conducted a randomized, double-blind, placebo-controlled phase II clinical trial that confirmed that ESWT treatment resulted in a significant reduction in UAB symptoms and a trend towards decreased PVR (Table 3) [82].

### 6.5. Surgery

#### 6.5.1. Latissimus Dorsi Detrusor Myoplasty (LDDM)

A meta-analysis conducted by Forte et al. summarized the efficacy of LDDM, with the majority of 58 patients who underwent LDDM being able to return to spontaneous voiding and having a PVR of less than 100 mL [144]. However, only single-arm clinical trials without control groups have confirmed these findings. The complexity of LDDM is also a concern, having a mean operative time of up to 536 ± 22 min.

#### 6.5.2. Others

Recently, several bladder reconstruction and nerve grafting procedures have been reported to improve outcomes. Agarwal et al. found that rectus abdominis detrusor myoplasty (RADM) effectively reduced the PVR, increased the urinary flow rate, and improved bladder contractility and detrusor pressure [145]. Similarly, Thorner et al. observed significant improvements in Q_max_ and PVR over a 1-year follow-up period with the use of reduction cystoplasty (RC) in a specific population with impaired detrusor contractility (IDC), particularly in PVR, which was reduced by 65–100% [146]. Lin et al. compared the S1 ventral root (VR) of the unilateral proximal anastomosis to the distal ends of the S2 and S3 ventral roots to treat atonic bladder caused by S2–S5 injury. Seven out of nine patients were followed up satisfactorily for 8–12 months after surgery, and the mean PVR decreased from 186.0 ± 35.0 mL to 43.0 ± 10.0 mL after the treatment [147].

### 6.6. Stem Cell Therapy

Stem cells possess self-renewal properties and can differentiate into various cell types under different conditions. Stem cell therapy has been used to treat various diseases. DU is caused by the loss of a detrusor or decreased contractility owing to a variety of etiological factors. Therefore, the injection of stem cells into the bladder wall for the treatment of DU is a promising therapeutic approach that has been used in several preclinical and clinical studies with promising results.

Teraoka et al. reported an improvement in maximum intravesical pressure (*p* = 0.009) and PVR (*p* = 0.011) in a rat cryoinjury model by increasing the expression of vascular endothelial growth factor and hepatocyte growth factor through adipose-derived stem cell sheet implantation [148]. Human embryonic stem cell-derived multipotent mesenchymal stem/stromal cells (M-MSCs) have been found to enhance the voiding function in diabetic DU rats by inhibiting bladder tissue apoptosis via paracrine effects [149]. Additionally, M-MSCs attenuated fibrosis and apoptosis to improve voiding function in atherosclerotic chronic bladder voiding in ischemic rats [150]. Recently, Sun et al. reported the use of human urine-derived stem cells (hUSCs) that were induced to differentiate into Cajal-like cells (ICC-LCs) and modified by the application of lentiviral vectors, which may play a role in the treatment of UAB [151]. In addition to the direct intravesical injection of stem cells, the introduction of exogenous stem cell factor (SCF) may improve voiding dysfunction caused by diminished numbers of interstitial cells of Cajal (ICC) in DU [152]. Shin et al. conducted a comprehensive review of animal experiments using stem cells in DU [153].

The case described by Levanovich et al. was the first example of a patient with UAB undergoing stem cell therapy [154]. This patient experienced a reduction in maximum bladder capacity from 844 mL to 663 mL during a 1-year follow-up period of treatment with autologous muscle-derived stem cells (AMDCs). The patient voided small amounts of urine and did not report any adverse events. Gilleran et al. discovered that 11 out of 19 patients (58%) who received intramuscular injections of AMDCs reported a global response assessment (GRA) of ≥5 [155]. This indicates a slight or significant improvement in UAB symptoms. Recently, Coelho et al. reported on a 54-year-old male patient who underwent intravesical injections of autologous mesenchymal stromal cells (AMSCs) [156]. The patient demonstrated a significant reduction in PVR from 800 mL to 20 mL as well as marked improvements in the maximum flow rate from 2 mL/s to 23 mL/s, maximum detrusion pressure from 21 cmH_2_O to 46 cmH_2_O, and BCI from 31 to 161. Another clinical study conducted by the same team, which involved nine patients, demonstrated that stem cell therapy derived from AMSCs was capable of increasing the maximal flow rate from 7.22 ± 1.58 mL/s to 13.56 ± 1.17 mL/s [157]. Additionally, it reduced the PVR from 420.00 ± 191.41 mL to 118.33 ± 85.51 mL, as well as reducing or eliminating the requirement for the daily use of ICs. Detailed data from these clinical trials are presented in Table 4. In conclusion, these initial case reports and single-arm clinical trials suggest that stem cell therapy is a promising treatment option for DU. However, further RCTs are necessary to establish the efficacy and safety of stem cell therapy.

### 6.7. Bladder Replacement Alternatives

With the progress in industrial technology, an increasing number of research teams are concentrating on manufacturing “artificial bladders” or similar products to reinstate the contractility of the urethral muscle or assist in urination. Lee et al. described a wireless, fully implantable, and scalable electronic complex, which was demonstrated in an animal model to monitor bladder function and provide electrical stimulation to induce urination based on a feedback control system [158]. Our team proposed an implantable magnetic soft robotic bladder (MRB) capable of applying mechanical compression directly to the bladder, thereby assisting the voiding process, in a UAB model pig [159]. Peh et al. devised a closed-loop pelvic neurostimulation system that could deliver varying stimulation currents by sensing bladder pressure signals and achieving a more than 75% success rate [160]. Various treatment options for UAB are currently being investigated, including neurostimulation, muscle stimulation, optogenetic technology, shape-memory alloys, and hydrogels. Holmes-Martin et al. presented a more complete review of adjunctive electronic devices that could be used for UAB treatment [161]. These devices generally have a closed-loop system that first monitors the intra-bladder pressure via sensors and then completes the voiding process via different technical routes. The two areas requiring further attention are: (1) accomplishing long-term device implantation and (2) achieving implantable device-assisted coordinated detrusor–sphincter movement. However, once severe bladder fibrosis occurs, achieving the intended results of DU treatment with drugs or surgery is difficult. Therefore, it is important to identify reliable bladder replacement options.

## 7. Future Directions and Prospects

In recent years, there have been various research developments in the domain of UAB/DU, encompassing the ICS definition of UAB, along with several RCTs that have established the efficacy of different drugs and treatments. In line with the suggested agenda for subsequent research presented by the International Congress on Underactive Bladder (CURE-UAB) [162] and the ICI-RS Think Tank [9], we propose that based on the current literature, there remain several unresolved concerns surrounding UAB that require further investigation: (1) There is no clear urodynamic definition of DU that is set with full consideration of the patient’s age, sex, and etiology. (2) The relationship between UAB symptoms and urodynamic DU should be established. (3) Prospective cohort studies are required to elucidate the natural history of UAB. (4) Designing reliable questionnaires, exploring non-invasive diagnostic methods for UAB, and validating them in large-scale prospective cohorts are necessary for the screening and early identification of UAB. (5) Multicenter large-scale RCTs need to be conducted to establish the safety and efficacy of new drugs or treatments, such as TAC-302, ESWT, and stem cell therapies. (6) Drug targets based on pathophysiological mechanisms including bladder ischemia, oxidative stress, inflammation, and fibrosis need to be investigated. (7) Research and development of bladder replacement devices is encouraged. In addition to the aforementioned research priorities, enhancing health education for patients is recommended, particularly for key populations such as individuals with neurological conditions and diabetes, to avert injuries in the upper urinary tract.

## 8. Materials and Methods

We used the keywords (“underactive bladder” OR “detrusor underactivity” OR “acontractile bladder” OR “detrusor failure” OR “detrusor areflexia” OR “atonic bladder” OR “impaired bladder contractility”) to search all the literature published from January 1950 to August 2023. Our search resulted in a total of 3540 relevant articles, which we retrieved. All original articles, reviews, commentaries, editorials, and case reports were included. After reading the titles and abstracts, a total of 3023 non-English literature, conference abstracts, duplicates and unrelated to DU/UAB were excluded. After reading the full text of the remaining 517 papers, we reviewed and screened 148 papers related to the definition, incidence, etiology, risk factors, urodynamic features, diagnosis, and treatment of DU/UAB for inclusion in this review. In addition, four studies in the reference lists of the selected studies were screened.

## 9. Limitations

This paper still has some limitations. Firstly, the studies included have small sample sizes or low qualities; thus, reporting bias should be considered when interpreting some of the findings. Furthermore, the review’s broad scope prevented any clear definition of the inclusion criteria for different literature aspects. Hence, notwithstanding that we explicated the figures and rationales for the integration of the articles at every stage of screening procedure, some degree of selection bias remains inescapable. In the future, systematic reviews or meta-analyses with a more specific focus should be conducted to provide better evidence for clinical diagnoses and treatment.

## 10. Conclusions

UAB, a prevalent and multi-etiological condition of the lower urinary tract, has not been sufficiently studied. Encouraging progress has been made in the field of UAB in recent years. The ICS defines UAB based on clinical symptoms, which will help identify patients with potential DU. The urodynamic definition of DU in various study groups generally considers sex differences and distinguishes DU from BOO. Moreover, several questionnaires and non-invasive diagnostic methods have been developed to detect UAB at an early stage. It is equally important to understand the etiology of DU and establish appropriate animal models to investigate its pathophysiological mechanisms. Preliminary clinical trials have validated the effectiveness of new drugs and treatments, including TAC-302, ESWT, and stem cell therapy for the management of UAB/DU. However, confirmation through large-scale multicenter RCTs is necessary. Future research is required to enhance the understanding of UAB/DU and establish a theoretical foundation for developing new therapeutic interventions.

## Figures and Tables

**Table 1:** Urodynamic diagnosis criteria for detrusor underactivity.

Author (Year)	Size (n)	Age (y)	Diagnosis Criteria for DU
Male			
Kaplan et al. (1996) [69]	137	18–50	P_det_@Q_max_ < 45 cmH_2_O, Q_max_ < 12 mL/s
Fusco et al. (2001) [70]	541	26–89	P_det_@Q_max_ < 30 cmH_2_O, Q_max_ < 12 mL/s
Nitti et al. (2002) [71]	85	18–45	BOOI < 20, Q_max_ < 12 mL/s
Wang et al. (2003) [72]	90	18–50	P_det_@Q_max_ < 30 cmH_2_O, Q_max_ < 15 mL/s
Abarbanel et al. (2007) [73]	82	>70	P_det_@Q_max_ < 30 cmH_2_O, Q_max_ < 10 mL/s
Jeong et al. (2012) [74]	632	>65	BCI < 100
Hoag et al. (2015) [75]	25	Mean: 59.2 (range, 19–90)	BCI < 100 and absence of identifiable BOO
Gammie et al. (2016) [76]	129	Median: 63	BCI < 100, BOOI < 20, BVE < 90%
Lee et al. (2016) [77]	111	Mean: 65.3 ± 9.2	BCI < 100 and BOOI < 20
Uren et al. (2017) [78]	29	Mean: 64 (range, 27–88)	BCI < 100 and BOOI < 20
Matsukawa et al. (2020) [79]	145	Mean: 69.5 ± 9.9	BCI ≤ 100 and BOOI ≤ 40
Namitome et al. (2020) [80]	454	Median: 70 (IQR, 65–76)	BCI < 100
Matsukawa et al. (2023) [81]	212	72.2	BCI ≤ 100 and BOOI ≤ 40
Shen et al. (2023) [82]	7	- *	BCI < 100 and BOOI < 20
Female			
Abarbanel et al. (2007) [73]	99	>70	Q_max_ < 10 mL/s, P_det_@Q_max_ < 30 cmH_2_O
Jeong et al. (2012) [74]	547	>65	P_det_@Q_max_ ≤ 10 cmH_2_O and Q_max_ ≤ 12 mL/s
Hoag et al. (2015) [75]	54	Mean: 59.2 (range, 19–90)	BCI < 100 and absence of identifiable BOO
Gammie et al. (2016) [76]	308	Median: 55	P_det_@Q_max_ ≤ 20 cmH_2_O and Q_max_ < 15 mL/s
Uren et al. (2017) [78]	15	Mean: 64 (range, 27–88)	P_det_@Q_max_ ≤ 20 cmH_2_O and Q_max_ < 15 mL/s
Shen et al. (2023) [82]	4	- *	P_det_@Q_max_ < 20 cmH_2_O and Q_max_ < 15 mL/s

P_det_@Q_max_: detrusor pressure at maximum flow; Q_max_: maximum flow rate; BCI: bladder contractility index; BOOI: bladder outlet obstruction index; BVE: bladder voiding efficiency. BCI = P_det_@Q_max_ + 5Q_max_. BOOI = P_det_@Q_max_ − 2Q_max_. BVE = (voided volume/total bladder capacity) × 100. * This study did not calculate patient age by gender.

**Table 2:** Recent clinical trials of pharmacotherapy for detrusor underactivity.

Author (Year)	Design	Treatment	Size (n)	Age (y)	Treatment Method	Safety	Efficacy (Intervention vs. Placebo)
Yoshida et al. (2022) [121]	RCT	TAC-302	TAC-302: 52 Placebo: 24	Median 74.0	Received oral 200 mg TAC-302 or placebo twice daily for 12 weeks.	AEs, serious AEs, and AEs leading to dose interruption were similar between groups	BCI (SD) for males: 12 wk: 75.2 (21.1) vs. 60.5 (16.7), *p* = 0.02; PIP1 (SD) for females: 12 wk: 29.4 (9.4) vs. 25.5 (9.6), *p* = 0.16; BVE for both sexes: 12 wk: 18.41% vs. 2.88%, *p* = 0.03 No significant differences in the mean number of micturition per 24 h, number of urgency episodes per 24 h, and total OABSS *
van Till et al. (2022) [112]	RCT	ASP8302	ASP8302: 65 Placebo: 70	62.8 (11.1) 61.1 (13.0)	Received 4-week oral once-daily administration of 100 mg ASP8302 or matching placebo.	AEs were similar between study groups	Median change in PVR_C2_ (Q1, Q3): 4 wk: −40.0 (−125.0, 25.0) vs. −35.0 (−130.0, 40.0), *p* = 0.960; Median change in BVE_C2_ (Q1, Q3): 4 wk: 2.6 (−7.1, 12.7) vs. 5.1 (−8.1, 14.5), *p* = 0.812 In males, ASP8302 showed improvement of symptoms and functional parameters.
Matsukawa et al. (2021) [116]	Prospective comparative study	Tadalafil Silodosin	Tadalafil: 59 Silodosin: 67	70.5 69.7	Received tadalafil 5 mg/day or silodosin 8 mg/day for 12 months	Not reported	Mean beneficial change (Tadalafil vs. Silodosin): Q_max_ (mL/s): 3.0 ± 3.0 vs. 1.7 ± 1.4, *p* < 0.001 BCI: 19.7 ± 17.7 vs. 6.0 ± 12.5, *p* < 0.001 PVR (mL): 20 (–6–70) vs. 16 (–3–54), *p* = 0.56

RCT: randomized clinical trial; AE: adverse event; BCI: bladder contractility index, P_det_@Q_max_ + 5Q_max_. PIP1: Projected isovolumetric pressure 1; SD: standard deviation; PVR_C2_: PVR after standardized bladder filling measured by catheterization; BVE_C2_: bladder voiding efficiency after standardized bladder filling. * This article examined the effects of TAC-302 on UAB and OAB but did not divide the subgroups by disease type.

**Table 3:** Recent randomized clinical trials of nerve electrical stimulation therapy and extracorporeal shock wave therapy for detrusor underactivity.

Author (Year)	Treatment	Size (n)	Age (y)	Treatment Method	Safety	Efficacy (Intervention vs. Placebo)
Liao et al. (2023) [134]	IVES	IVES: 38 Placebo: 38	44.6 47.0	Pulse width of 0.2 ms, frequency of 20 Hz, intensity max tolerated; once a day for 30 min, 5 days a week for a total of 4 weeks	4 UTI in IVES and 2 UTI in placebo, *p* = 0.67; No severe AEs were reported	PVR changes for IVES: 2 wk: −64.1 (87.1) mL vs. −9.6 (90.9) mL, *p* < 0.01 4 wk: −97.1 (107.5) mL vs. −10.5 (86.7) mL, *p* < 0.01 No significant differences in the number of 24-h CIC procedures, PPBC-S, or AUA-SI-QoL score between the groups.
Kajbafzadeh et al. (2016) [136]	IFES	IFES: 18 Control:18	8.4 (2.4) 9.2 (2.5)	20 min twice a week, 15 courses; 4 kHz carrier frequency and a beat frequency sweep at 5–55 Hz	No side-effects reported during and after IFES	At the end of treatment courses: NOV (SD): 6.3 (1.4) vs. 4.7 (1.3) times/day, *p* < 0.002 BC (SD): 238 (58) vs. 366 (67) mL, *p* < 0.001; Maximum urine flow: 22.1 (6.8) vs. 11.8 (5.7) mL/s, *p* = 0.001; PVR: 14.1 (5.8) vs. 49 (23.3), *p* = 0.03. 1-year follow-up: BC (SD): 227 (86) vs. 344 (127) mL, *p* = 0.016; Maximum urine flow: 21 (8.3) vs. 12.8 (4.8) mL/s, *p* = 0.001; PVR: 22.5 (10.3) vs. 44.1 (39), *p* = 0.03.
Shen et al. (2023) [82]	ESWT	ESWT: 6 Placebo: 5	62.4 61.2	4 pulses/s, 2500 shocks, max energy flow density of 0.25 mJ/mm^2^, once a week for 6 wk	No hematuria, pain, or infection in the two groups reported.	Improvement in PVR (95%CI): 4 wk: 157.8 mL (−380.1,64.4) vs. −6.6 mL (−178.1, 164.9), *p* = 0.116 12wk: 77.5 mL (−242.1, 87.1) vs. 81.8 mL (−137.2, 300.7), *p* = 0.056 reduction in the UAB-Q score (95%CI): 4 wk: −4.3 (−9.1, 0.4) vs. 0.4 (−1.8, 1.0), *p* = 0.025 12wk: −2.7 (−5.5, 0.1) vs. −0.2 (−3.5, 3.1), *p* = 0.091

IVES: intravesical electrical stimulation; UTI: urinary tract infection; PPBC-S: Patient Perception of Bladder Condition Scale; AUA-SI-QoL: American Urological Association Symptom Index Quality of Life; BCI: bladder contractility index = P_det_@Q_max_ + 5Q_max_; IFES: transcutaneous interferential electrical stimulation; NOV: number of voiding; SD: standard deviation; BC: bladder capacity; PVR: post-void residual; ESWT: extracorporeal shock wave therapy; UAB-Q: underactive bladder questionnaire.

**Table 4:** Clinical trials of stem cell therapy for detrusor underactivity.

Author (Year)	Treatment	Size (n)	Age (y)	Treatment Method	Safety	Efficacy (Intervention vs. Placebo)
Levanovich et al. (2015) [154]	AMDC	1	79	Injections of a total of 250 million AMDC in a total volume of 15 mL distributed throughout approximately 30 sites.	No treatment-related adverse events or side effects were reported	3 m after injection: first desire 711 to 365 mL; strong desire 823 to 450 mL; MCC: 844 to 663 mL. Using PGI-I questionnaire the patient reported “a little bit better”, while using GRA reported “moderate improvement” of UAB symptoms at 6 and 12 months.
Gilleran et al. (2021) [155]	AMDC	20	64.3 ±11.6	Injections of a total of 125 million AMDC in a total volume of 15 mL distributed throughout approximately 30 sites.	Minor AEs such as UTI, which resolved spontaneously without sequelae.	Patient-reported GRA: 3 m: 30% improved; 70% no change. 6 m: 50% improved; 45% no change; 5% worse. 12 m: 58% improved; 21% no change; 21% undetermined. PVR was trending downward while VE was trending upward.
Coelho et al. (2023) [156]	AMSC	1	54	2 × 10^6^ AMSCs were injected in the bladder trigone 60 days after fat collection, and a second injection was performed 30 days later.	No complications reported.	60 days after last transplant vs. before: Residual volume: 20 mL vs. 800 mL; Maximum Flow: 23 mL/s vs. 2 mL/s; max P_det_: 46 cmH_2_O vs. 21 cmH_2_O; BCI: 161 vs. 31; ICIQ-SF: 1 vs. 19.
Coelho et al. (2023) [157]	AMSC	9	65.66 ± 2.49	2 × 10^6^ AMSCs were injected in the bladder trigone 60 days after fat collection, and a second injection was performed 30 days later.	4 ecchymosis, 1 edema, 3 pains, 2 UTI, 1 hematuria, 1 dysuria	60 days after last transplant vs. before: Residual volume: 118.33 ± 85.51 mL vs. 420.00 ± 191.41 mL; *p* < 0.05 Maximum Flow: 11.56 ± 1.67 mL/s vs. 7.78 ± 0.76 mL/s; *p* < 0.05 max P_det_: 41.56 ± 5.75 cmH_2_O vs. 20.22 ± 8.29 cmH_2_O; *p* < 0.05 BCI: 100.56 ± 8.89 vs. 44.33 ± 4.85; *p* < 0.05 ICIQ-SF: 3.78 ± 0.78 vs. 11.44 ± 1.43; *p* < 0.05.

AMDC: autologous muscle derived cell; PGI-I: patient global impression of improvement; GRA: global response assessment; AE: adverse effects; UTI: urinary tract infection; GRA: global response assessment; PVR: post-void residual; VE: voiding efficiency. AMSC: autologous mesenchymal stromal cells; max P_det_: maximum detrusion pressure; BCI = P_det_@Q_max_ + 5Q_max_. ICIQ-SF: International Continence on Incontinence Questionnaire-Short Form.

## Data Availability

No new data were created or analyzed in this study. Data sharing is not applicable to this article.
